# Efficacy and safety of concomitant left atrial appendage closure and pulmonary vein isolation compared to left atrial appendage closure only – A meta-analysis

**DOI:** 10.1016/j.ijcha.2026.101988

**Published:** 2026-08-11

**Authors:** Isabel Rattka, Christine Poch, Tanja Mews, Constantin Friesacher, Niklas Jurik, Eimo Martens, Karl-Ludwig Laugwitz, Alexander Steger, Manuel Rattka

**Affiliations:** School of Medicine and Health, Department of Clinical Medicine—Clinical Department for Cardiology, University Medical Centre, Technical University of Munich, Munich, Germany

**Keywords:** Left atrial appendage closure, Pulmonary vein isolation, Concomitant, Stroke, Pericardial effusion, Peri-device leak

## Abstract

**Background:**

Atrial fibrillation (AF) is associated with an increased risk of ischemic stroke, primarily originating from the left atrial appendage (LAA). Percutaneous LAA closure (LAAC) reduces appendage-borne thromboembolism, while pulmonary vein isolation (PVI) restores sinus rhythm. Observational data on the combined one-stop procedure are heterogeneous, and data on its risk-benefit profile remain scarce. Thus, a meta-analysis was performed to compare the efficacy and safety of combined LAAC+PVI versus LAAC-only.

**Methods:**

A structured systematic search of PubMed, MEDLINE, Scopus and Web of Science was performed for studies comparing outcomes of AF patients undergoing combined LAAC+PVI or LAAC-only.

**Results:**

Fifteen studies reporting data from 335,013 patients (11,839 LAAC+PVI; 323,174 LAAC-only) were included. Combined LAAC+PVI was associated with significantly fewer systemic thromboembolisms at follow-up (OR = 0.76, 95% CI = 0.60–0.96, I^2^ = 1%) compared to LAAC-only. However, periprocedural pericardial effusion requiring drainage was significantly more frequent in the combined group (OR = 1.72, 95% CI = 1.26–2.34, I^2^ = 0%). Peri-device leaks were significantly less common immediately post-procedure (OR = 0.57, 95% CI = 0.35–0.92, I^2^ = 19%), but significantly more frequent on follow-up echocardiography (OR = 1.56, 95% CI = 1.14–2.12, I^2^ = 71%). Mortality, major bleeding, device-related thrombus, and pericardial effusion without drainage did not differ significantly between groups.

**Conclusion:**

Combined LAAC+PVI was associated with fewer systemic thromboembolisms than LAAC-only, but a higher periprocedural risk of pericardial effusion requiring drainage and divergent temporal behaviour of peri-device leaks. Randomized controlled trials are warranted to confirm these findings.

## 1 Introduction

Atrial fibrillation (AF) confers a substantially increased risk of ischemic stroke and systemic thromboembolism. In non-valvular AF, the left atrial appendage (LAA) represents the predominant source of atrial thrombi. [1], [2] Oral anticoagulation remains the established standard for stroke prevention. In patients in whom it is contraindicated or poorly tolerated, percutaneous LAA occlusion (LAAC) has emerged as a mechanical alternative. [1] Independently, pulmonary vein isolation (PVI) by catheter ablation has been established as the most effective rhythm-control strategy in symptomatic AF, reducing arrhythmia burden and improving quality of life. [2] LAAC addresses appendage-borne thromboembolism, while PVI addresses arrhythmogenesis itself. The two interventions are therefore mechanistically complementary and combining them in a single procedure has gained increasing attention.

Observational data suggest that the combined approach is feasible and may be associated with a reduction in ischemic events. [3] At the same time, signals of higher periprocedural complication rates and a distinct temporal behaviour of peri-device leaks (PDL) have been reported. [3], [4], [5] However, the available evidence remains heterogeneous, and it remains unresolved whether the combined procedure offers a clinical benefit over LAAC alone or merely trades one risk for another. To date, neither a randomized controlled trial nor a meta-analysis has confirmed these signals. [6]

We therefore hypothesized that combined LAAC and PVI is associated with a reduction in systemic thromboembolic events relative to LAAC alone, at the cost of a distinct periprocedural risk profile. To test this hypothesis, we performed a meta-analysis comparing the efficacy and safety of combined LAAC and PVI versus LAAC alone, with particular emphasis on systemic thromboembolism, periprocedural pericardial effusion, and the temporal evolution of peri-device leaks.

## 2 Methods

### 2.1 Data sources and study selection

**Fig. 1:**
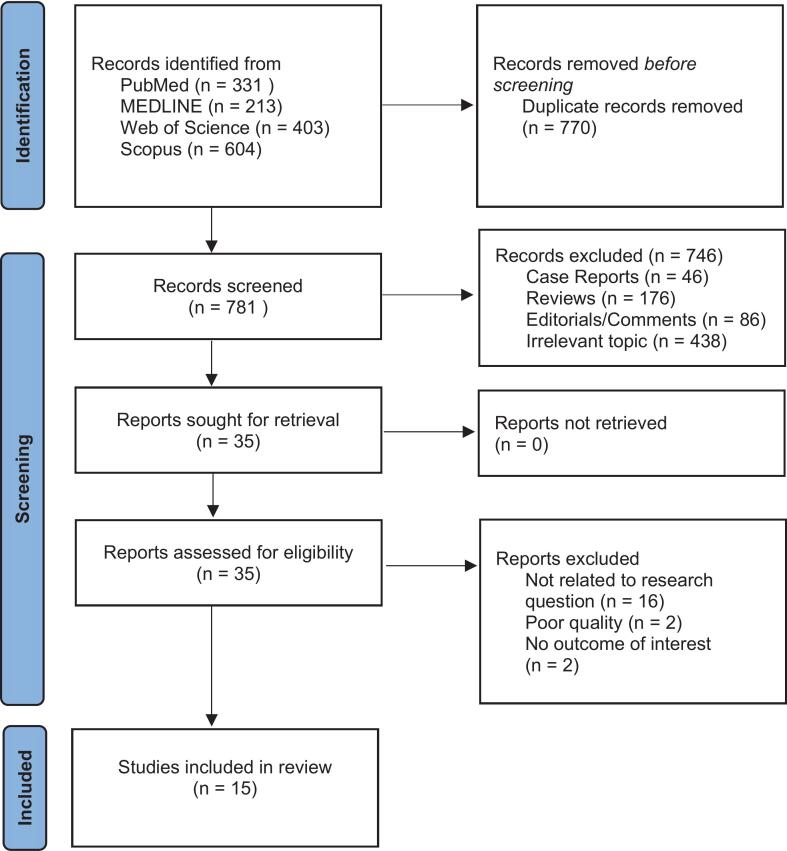
PRISMA flow diagram Out of 1551 identified studies and after application of the inclusions and exclusion criteria fifteen studies were included in the quantitative synthesis.

A comprehensive literature search was performed through PubMed, MEDLINE (via PubMed), Scopus and Web of Science through May 23rd, 2026 for studies comparing outcomes of AF patients treated by either LAAC + PVI or LAAC-only using the keywords “left atrial appendage closure” and “pulmonary vein isolation” and “atrial fibrillation”. The complete search strategy is shown in the supplements. We searched for randomized controlled studies, observational studies, and register/database-based studies, without language restrictions. Conference abstracts, case reports, review articles, and posters were excluded, as these sources typically lack sufficient methodological detail to allow reliable data extraction and quality assessment. The flowchart illustrating the literature search strategy is shown in Fig. 1.

### 2.2 Data extraction and study quality

Study selection, data extraction and quality assessment were performed as described in our previous meta-analyses. [7], [8] In short, two investigators independently reviewed all the articles, selected eligible studies and extracted relevant data. In case of any discrepancies, a third reviewer made the final decision. Data extraction used standardized extraction forms including information on authors, country of origin, publication year, number of patients per group, number of complications at the time of the procedure and during follow-up (any access site complication, pericardial effusion with/without drainage, transient ischemic attack (TIA) and/or stroke, any reported arterial thromboembolism, death, minor/major bleeding), number of patients with device related problems (device-related thrombus (DRT), peri-device leakage (PDL), and baseline characteristics (age, gender, percentage of patients with paroxysmal AF, number of patients with a history of TIA/stroke, arterial hypertension and diabetes mellitus, HAS-BLED score, and CHA_2_DS_2_-VASc Score). The study quality was assessed as detailed by the NIH Quality Assessment Tool and studies were rated as being of either “good”, “fair” or “poor” quality. [9] Studies rated as being of “poor” quality were excluded from further analysis. As all analyses were based on previous studies, neither patient consent nor ethical committee approval was required for our study. This meta-analysis has been pre-registered at PROSPERO [CRD420261385011].

### 2.3 Primary outcomes

We sought to compare the efficacy and safety of LAAC+PVI versus LAAC-only in patients with AF. The primary efficacy outcome was systemic thromboembolism (ischemic stroke, TIA, or systemic arterial embolism) during follow-up. Secondary efficacy outcomes included device-related thrombus at the end of follow-up and peri-device leak, assessed both immediately following the procedure and at the end of follow-up. The primary safety outcome was periprocedural pericardial effusion requiring drainage. Secondary safety outcomes were periprocedural pericardial effusion not requiring drainage, periprocedural systemic thromboembolism, periprocedural groin complications, periprocedural death, all-cause death during follow-up, and bleeding during follow-up.

### 2.4 Statistical analysis

The statistical methodology applied in this meta-analysis followed the approach used in our previous meta-analyses. [7], [8] In brief, data were collected in a Microsoft Excel® spreadsheet and outcome measures were calculated. If in the included studies only median values with interquartile ranges were reported, means and standard deviations were calculated by making use of the Box-Cox method, if possible. [10] Meta-analyses were performed on binary endpoints. The data are reported as absolute frequencies and/or percentages. The random effects model was used to combine the estimates from the different studies. This was preferred over the fixed effects model since the random effect model gives a more conservative estimate. For binary endpoints the Mantel-Haenszel method was used, and the odds ratio (OR) including 95% confidence interval is reported as effect measure. For the assessment of heterogeneity among studies, the statistical characteristic I^2^ is reported. Forest plots are used for graphical representation of results, which included all individual results of the considered studies separately, as well as overall results. Additionally, funnel plots were created to evaluate the risk of publication bias. Meta-analyses were performed using Review Manager (RevMan) version 5.4 (The Cochrane Collaboration, Copenhagen, Denmark). All tests were two-tailed, and results with a *p*-value of <0.05 were considered statistically significant. The Preferred Reporting Items for Systematic Reviews and Meta-Analyses (PRISMA) statement was applied in this study. [11]

## 3 Results

### 3.1 Literature search, quality assessment and baseline characteristics

Based on our search strategy, 1551 studies were identified from the PubMed, MEDLINE, Scopus and Web of Science databases. After removal of 770 duplicates, exclusion of 308 studies (46 case reports, 176 reviews, 86 editorials) and a further 438 studies not related to the research question, 35 articles were assessed for eligibility. Finally, after exclusion of 16 articles not related to the research question, two studies of poor quality as per the NIH tool, and two studies that reported no outcome of interest, 15 studies were included in the quantitative synthesis (Fig. 1). [3], [4], [5], [12], [13], [14], [15], [16], [17], [18], [19], [20], [21], [22], [23] Based on the NIH Study Quality Assessment tool 12 studies had “good”, and 3 studies “fair” methodological quality (Table 1). A total of 335,013 patients were enrolled from the various studies, including 11,839 patients undergoing LAAC + PVI and 323,174 patients treated by LAAC-only. Evaluation of baseline characteristics showed that the average age was 77 years with 42% of patients being female. The mean CHA2DS2-VASc Score was 4.9 ± 1.5 and the HAS-BLED Score 3.0 ± 1.3. Detailed information on baseline characteristics is listed in Table 1. (See Table 2.)

**Table 1:** Summary of included studies.

Study	Country/Database	mono- / multicentric	NIH	No. of patients
Hao et al. 2025	China	monocentric	good	166
Khan et al. 2026	NIS DB	–	fair	201,015
Li et al. 2024	China	monocentric	good	153
Pasupula et al. 2023	NRD DB	–	fair	782
Motairek et al. 2025	TriNetX DB	–	fair	32,280
Zhu et al. 2023	China	monocentric	good	98
Preisendörfer et al. 2024	US	monocentric	good	216
Zhang et al. 2023	China	monocentric	good	361
Mo et al. 2020	China	monocentric	good	152
Guo et al. 2025	China	monocentric	good	424
Piccini et al. 2026	NCDR	–	good	96,968
Li et al. 2020	China	monocentric	good	107
Tam et al. 2025	Hong Kong/Singapore	multicentric	good	84
Zhang et al. 2026	China	multicentric	good	2032
Atasi et al. 2026	US	monocentric	good	175

DB, database; NIS, National Inpatient Sample; NRD, Nationwide Readmission Database; NCDR, national cardiovascular data registry; NIH, study quality as per NIH Quality Assessment Tool.

**Table 2:** Summary of patient characteristics.

Study	Group	No. patients	Age	Female	paroxAF	AHT	DM	Stroke/TIA	CHA_2_DS_2_-VASC	HAS-BLED
Hao et al. 2025	LAAC	83	69.8 ± 4.7	28	18	61	14	16	3.4 ± 0.2	2.2 ± 0.1
	LAAC+PVI	83	69.4 ± 3.9	33	23	64	15	17	3.5 ± 0.2	2.2 ± 0.1
Khan et al. 2026	LAAC	198,345	76.8 ± 7.7	42	N/A	87	17	N/A	N/A	N/A
	LAAC+PVI	2670	73 ± 8.4	40	N/A	84	15	N/A	N/A	N/A
Li et al. 2024	LAAC	51	69.7 ± 1.1	39	N/A	73	28	43	4.0 ± 1.5	N/A
	LAAC+PVI	102	70.6 ± 1.0	36	N/A	71	24	43	4.0 ± 3.2	N/A
Pasupula et al. 2023	LAAC	407	N/A	47	55	91	34	N/A	N/A	N/A
	LAAC+PVI	375	N/A	47	49	83	30	N/A	N/A	N/A
Motairek et al. 2025	LAAC	27,576	N/A	N/A	N/A	N/A	N/A	N/A	N/A	N/A
	LAAC+PVI	4704	N/A	N/A	N/A	N/A	N/A	N/A	N/A	N/A
Zhu et al. 2023	LAAC	47	64.9 ± 8.6	51	38	53	19	75	3.7 ± 1.0	3.6 ± 0.9
	LAAC+PVI	51	64.4 ± 9.4	51	31	61	24	73	3.8 ± 1.0	3.5 ± 0.8
Preisendörfer et al. 2024	LAAC	144	76.7 ± 6.9	41	56	95	27	19	4.4 ± 1.2	2.3 ± 0.7
	LAAC+PVI	72	70.2 ± 7.3	50	63	97	33	13	4.2 ± 1.1	2.2 ± 0.8
Zhang et al. 2023	LAAC	78	63.6 ± 9.2	40	24	56	19	54	3.9 ± 1.5	2.3 ± 1.2
	LAAC+PVI	283	63.7 ± 7.8	41	35	65	20	48	3.9 ± 1.5	2.3 ± 1.3
Mo et al. 2020	LAAC	76	71.4 ± 8.9	50	36	75	20	26	3.7 ± 1.4	3.4 ± 1.1
	LAAC+PVI	76	69.9 ± 7.9	49	49	74	18	30	3.6 ± 1.3	3.3 ± 1.1
Guo et al. 2025	LAAC	58	67.5 ± 8.7	40	9	66	26	62	5.2 ± 1.6	3.5 ± 0.7
	LAAC+PVI	366	63.9 ± 7.6	42	37	73	23	57	4.5 ± 1.5	3.3 ± 0.6
Piccini et al. 2026	LAAC	95,124	77.0 ± 7.4	41	N/A	N/A	N/A	N/A	5.0 ± 1.5	3.0 ± 1.3
	LAAC+PVI	1844	73.0 ± 7.4	40	N/A	N/A	N/A	N/A	4.0 ± 1.5	2.4 ± 0.9
Li et al. 2020	LAAC	46	67.1 ± 7.4	44	4	74	28	57	4.3 ± 1.1	3.6 ± 1.2
	LAAC+PVI	61	66.7 ± 9.2	59	7	82	26	64	4.2 ± 1.1	3.4 ± 1.1
Tam et al. 2025	LAAC	48	75.1 ± 6.8	25	33	N/A	N/A	N/A	4.0 ± 1.4	N/A
	LAAC+PVI	36	65.7 ± 7.8	33	36	N/A	N/A	N/A	2.4 ± 1.2	N/A
Zhang et al. 2026	LAAC	1016	68.8 ± 9.8	39	43	69	22	68	3.9 ± 1.9	2.4 ± 1.1
	LAAC+PVI	1016	68.9 ± 8.8	41	44	69	25	68	4.0 ± 1.8	2.4 ± 1.1
Atasi et al. 2026	LAAC	75	77.4 ± 6.5	33	59	81	29	13	4.0 ± 1.5	N/A
	LAAC+PVI	100	N/A	N/A	N/A	N/A	N/A	N/A	N/A	N/A

LAAC, left atrial appendage closure; PVI, pulmonary vein isolation; paroxAF, paroxysmal atrial fibrillation; AHT, arterial hypertension; DM, diabetes mellitus; TIA, transient ischemic attack.

### 3.2 Efficacy outcomes

**Fig. 2:**
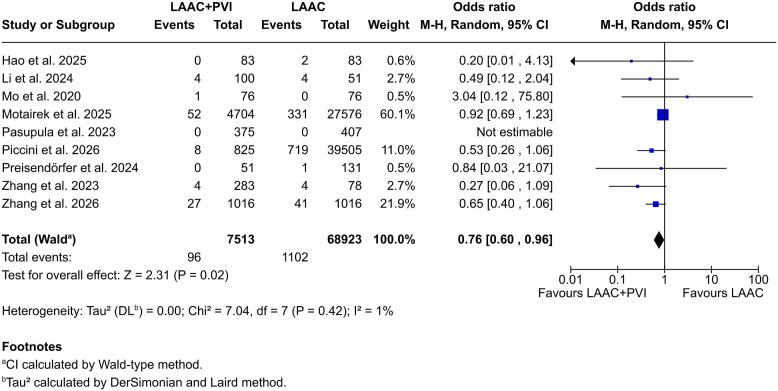
Forest plot – systemic thromboembolism, follow-up Systemic thromboembolism was less frequent in patients undergoing concomitant LAAC + PVI. LAAC, left atrial appendage closure; PVI, pulmonary vein isolation; CI, confidence interval.

**Supplementary Fig. S1:**
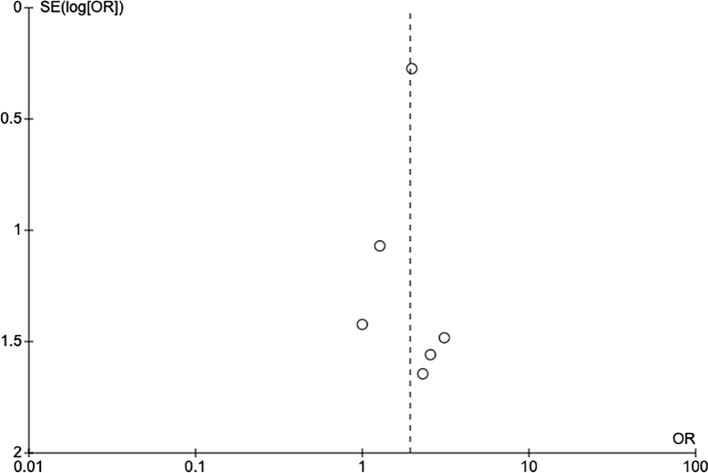
Funnel plot – systemic thromboembolism, follow-up.

The primary efficacy outcome was assessed by the number of systemic thromboembolisms at the end of follow-up including stroke/TIA and other reported systemic embolisms and could be extracted from nine studies. Remarkably, systemic thromboembolisms were significantly lower in patients undergoing LAAC+PVI compared to LAAC-only (OR = 0.76, 95% CI = 0.60–0.96, I^2^ = 1%; Fig. 2, Supplementary Fig. 1).

**Supplementary Fig. S2:**
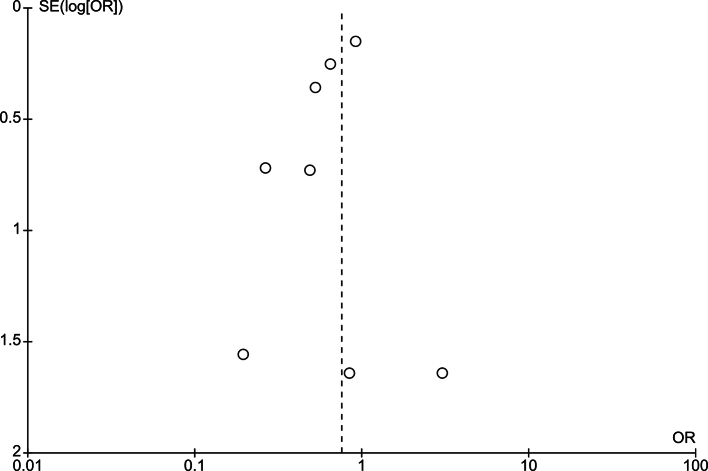
Forest plot – device related thrombus, follow-up.

**Supplementary Fig. S3:**
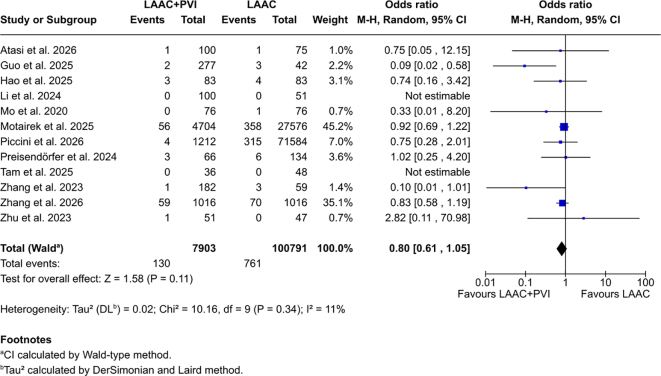
Funnel plot – device related thrombus, follow-up.

Device-related thrombus, reported across 12 studies, did not differ significantly between groups (OR = 0.80, 95% CI = 0.61–1.05, I^2^ = 11%; Supplementary Figs. 2 and 3).

**Fig. 3:**
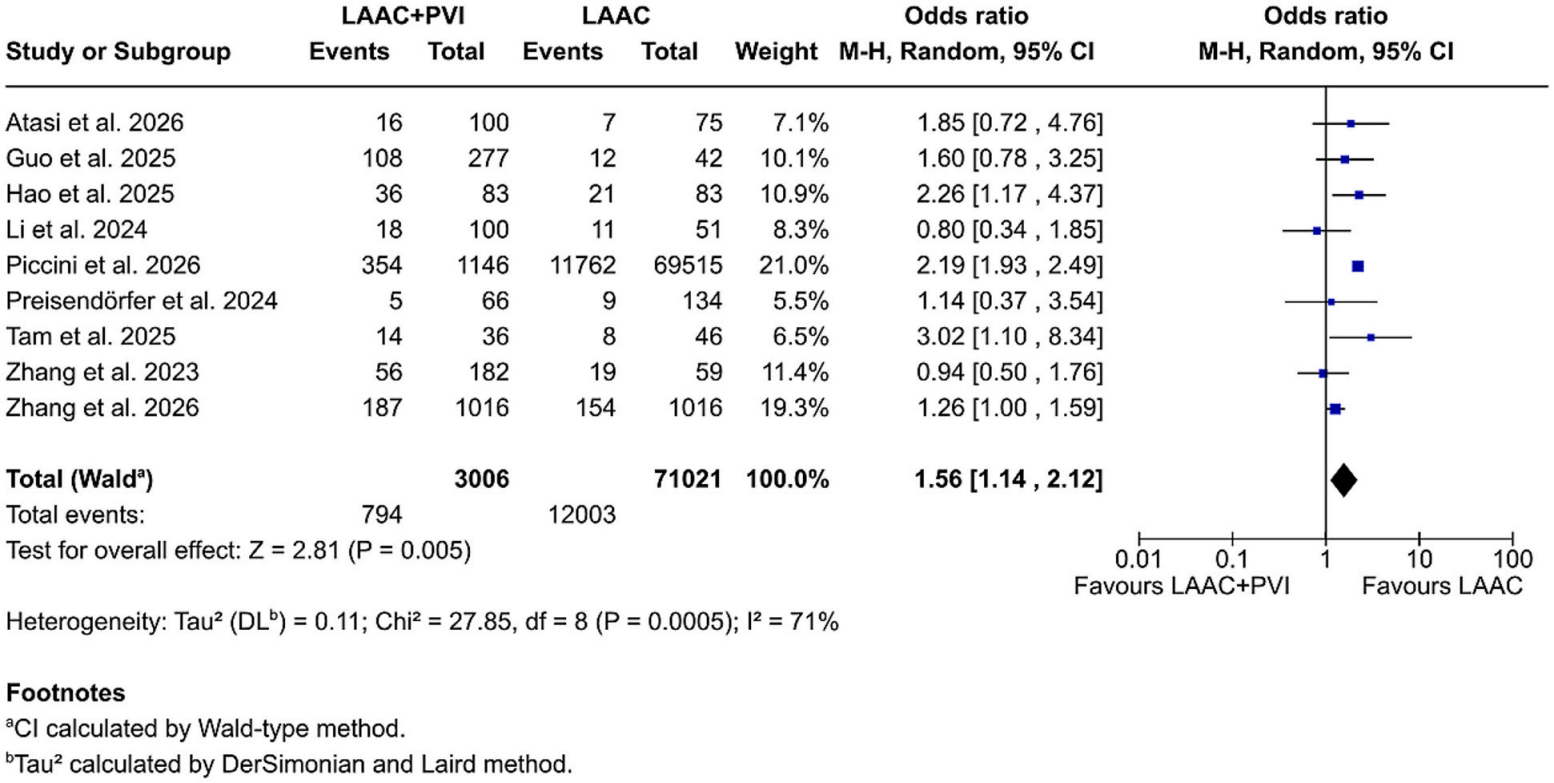
Forest plot – peri-device leaks, follow-up Overall, peri-device leaks during follow-up were observed significantly more often in the LAAC + PVI group. LAAC, left atrial appendage closure; PVI, pulmonary vein isolation; CI, confidence interval.

**Fig. 4:**
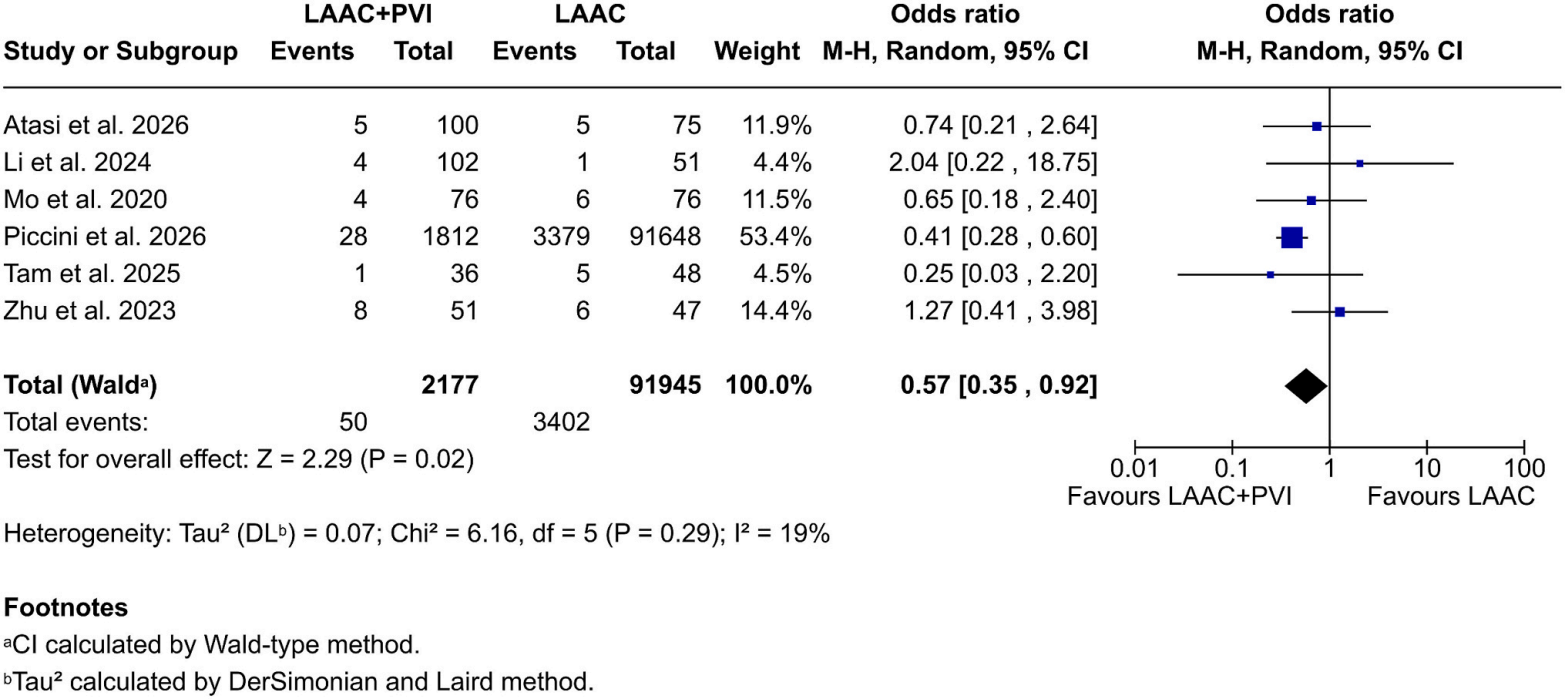
Forest plot – peri-device leaks, periprocedural Periprocedural device-leaks were less common after LAAC + PVI compared to LAAC-only. LAAC, left atrial appendage closure; PVI, pulmonary vein isolation; CI, confidence interval.

**Supplementary Fig. S4:**
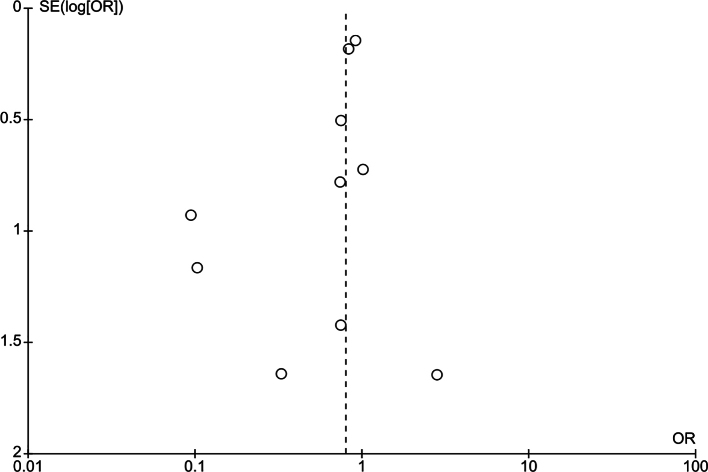
Funnel plot – peri-device leaks, follow-up.

**Supplementary Fig. S5:**
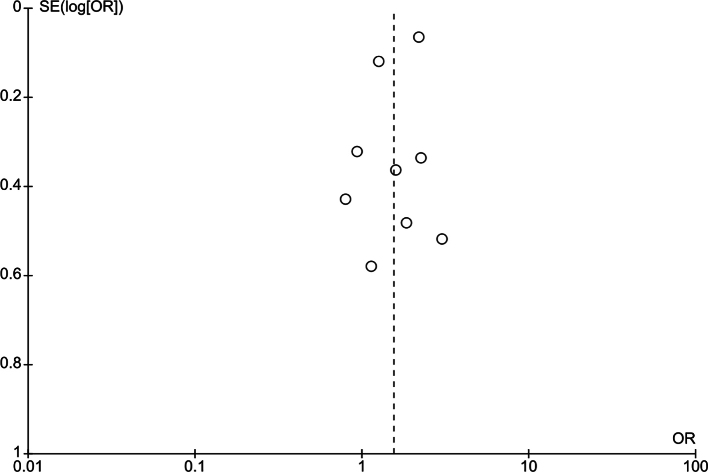
Funnel plot – peri-device leaks, periprocedural.

Interestingly, patients undergoing concomitant LAAC+PVI were more likely to show peri-device leaks during control echocardiography during follow-up as compared to the LAAC-only group (OR = 1.56, 95% CI = 1.14–2.12, I^2^ = 71% Fig. 3, Supplementary Fig. 4). This contrasts with the observation that, immediately post-procedure, PDLs occurred significantly less frequently in the LAAC+PVI group (OR = 0.57, 95% CI = 0.35–0.92, I^2^ = 19%; Fig. 4, Supplementary Fig. 5).

### 3.3 Safety outcomes

**Fig. 5:**
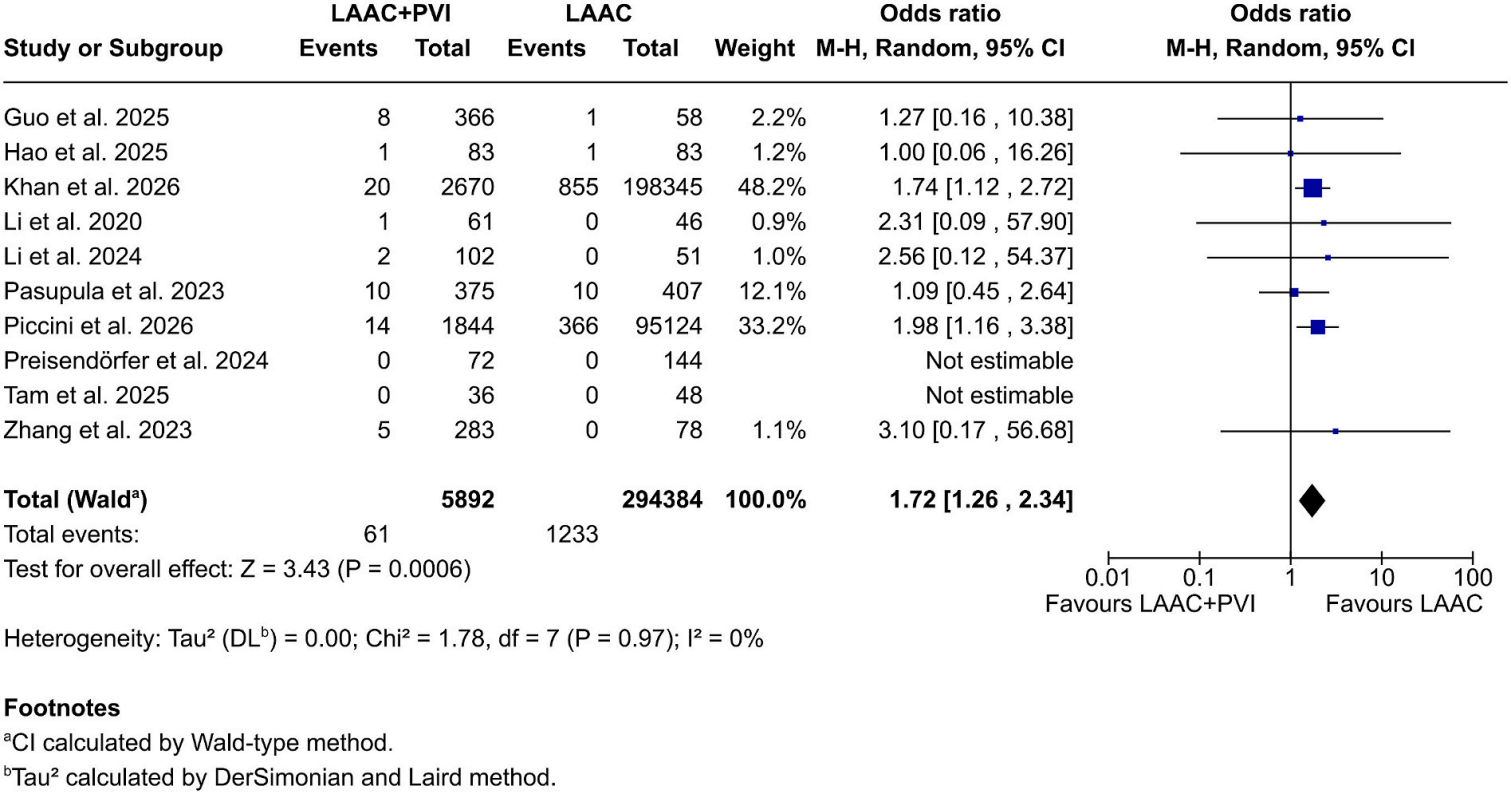
Forest plot – pericardial effusion requiring drainage Pericardial effusion requiring drainage was observed significantly more often following combined PVI + LAAC than LAAC alone. LAAC, left atrial appendage closure; PVI, pulmonary vein isolation; CI, confidence interval.

**Fig. 6:**
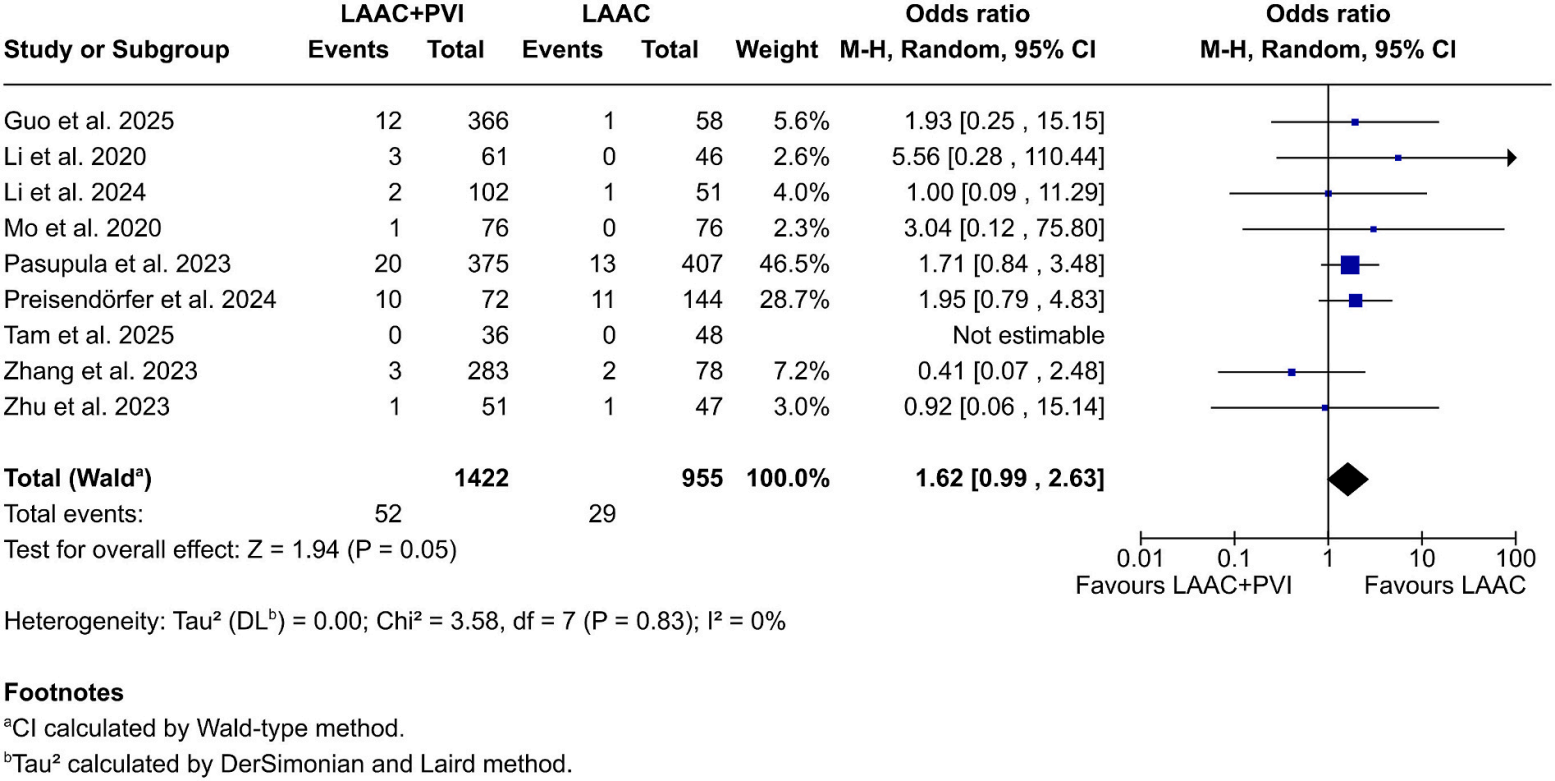
Forest plot – pericardial effusion without drainage For pericardial effusion without drainage there was no significant difference between the groups. CI, confidence interval.

**Supplementary Fig. S6:**
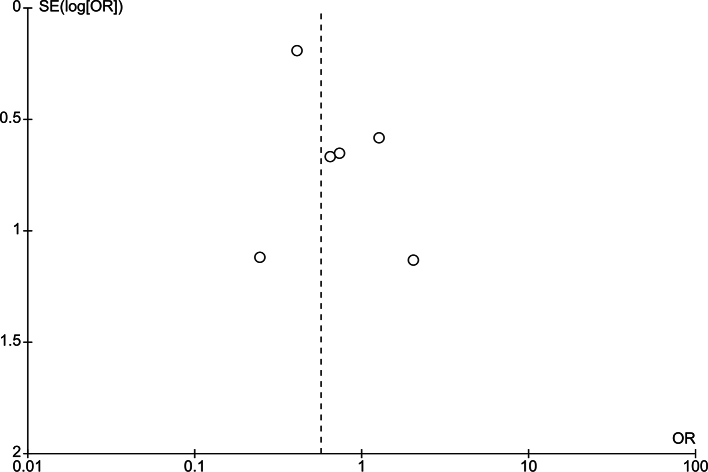
Funnel plot – pericardial effusion requiring drainage.

**Supplementary Fig. S7:**
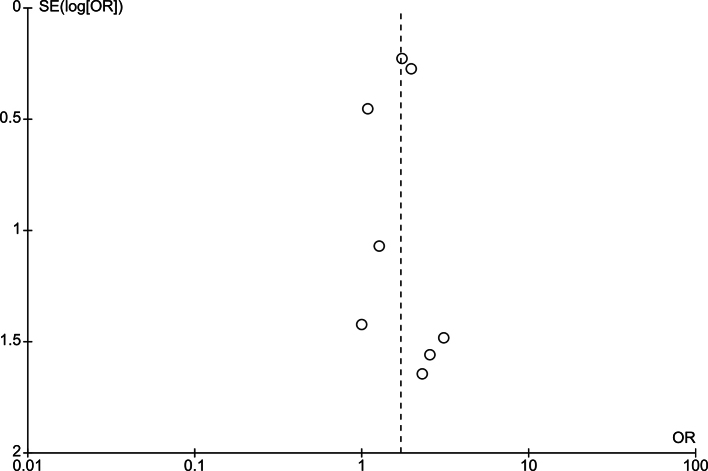
Funnel plot – pericardial effusion without drainage.

**Supplementary Fig. S8:**
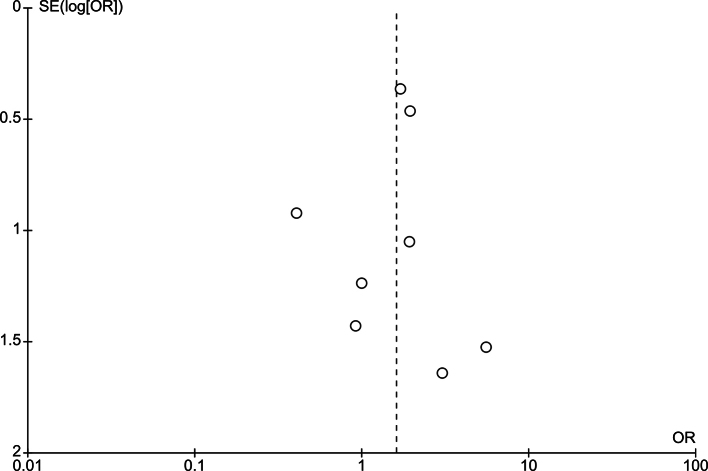
Forest plot – systemic thromboembolism, periprocedural.

**Supplementary Fig. S9:**
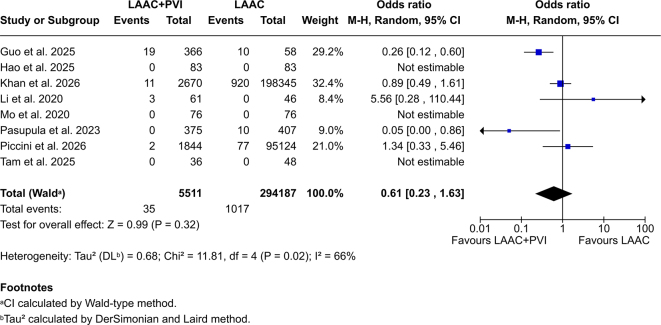
Funnel plot – systemic thromboembolism, periprocedural.

**Supplementary Fig. S10:**
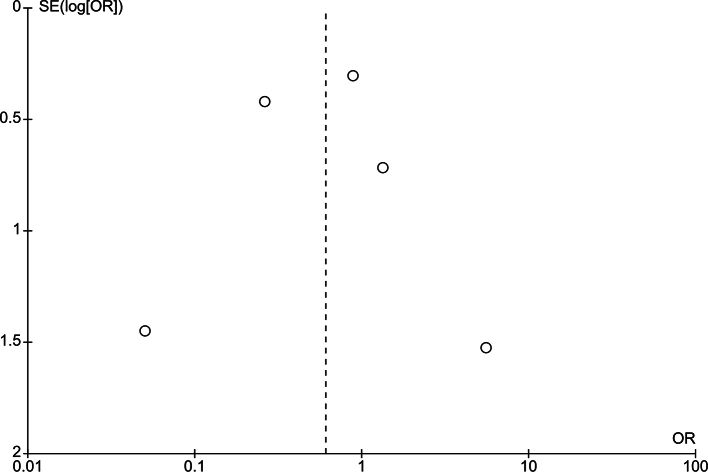
Forest plot – groin complications, periprocedural.

**Supplementary Fig. S11:**
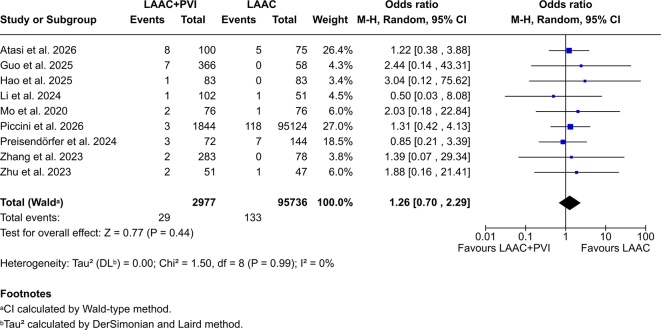
Funnel plot – groin complications, periprocedural.

**Supplementary Fig. S12:**
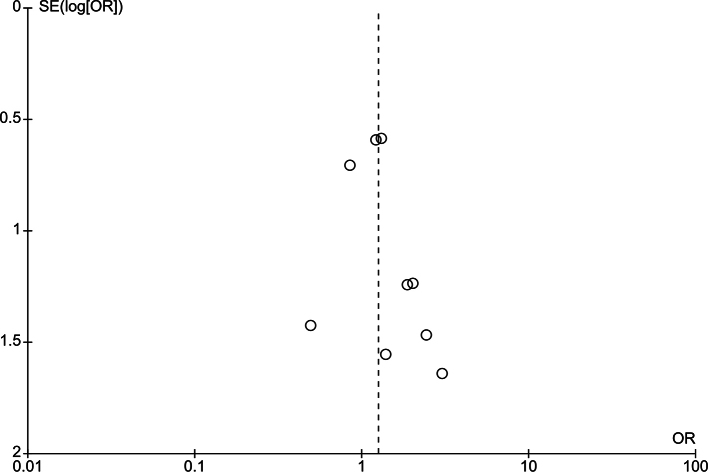
Forest plot – death, periprocedural.

**Supplementary Fig. S13:**
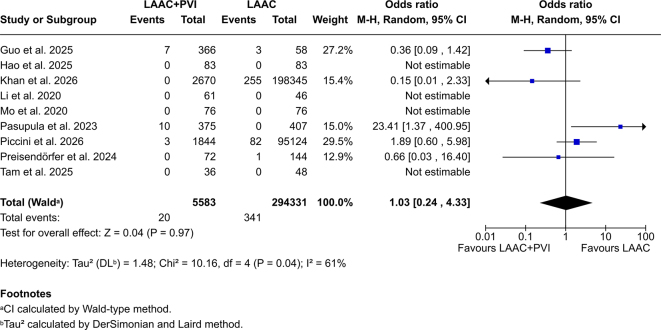
Funnel plot – death, periprocedural.

**Supplementary Fig. S14:**
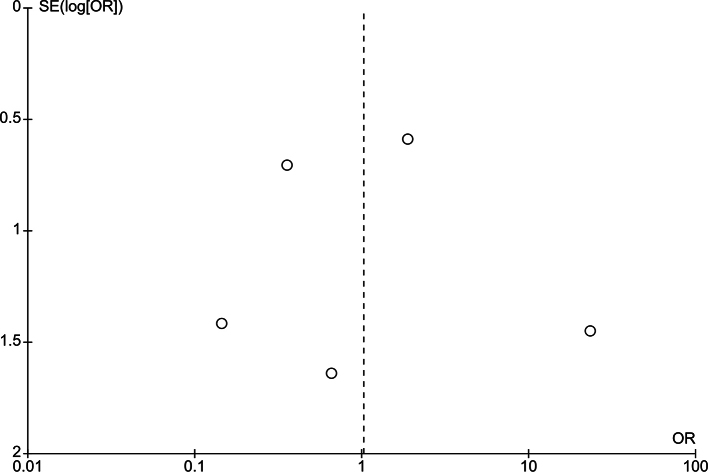
Forest plot – death, follow-up.

**Supplementary Fig. S15:**
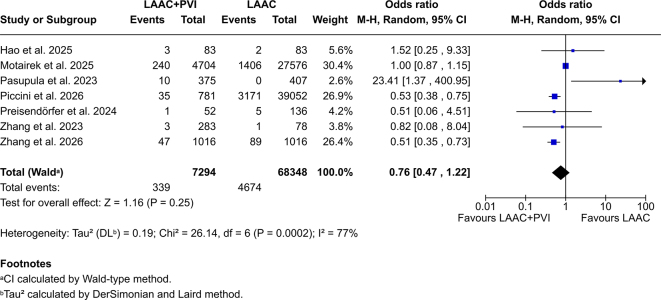
Funnel plot – death, follow-up.

**Supplementary Fig. S16:**
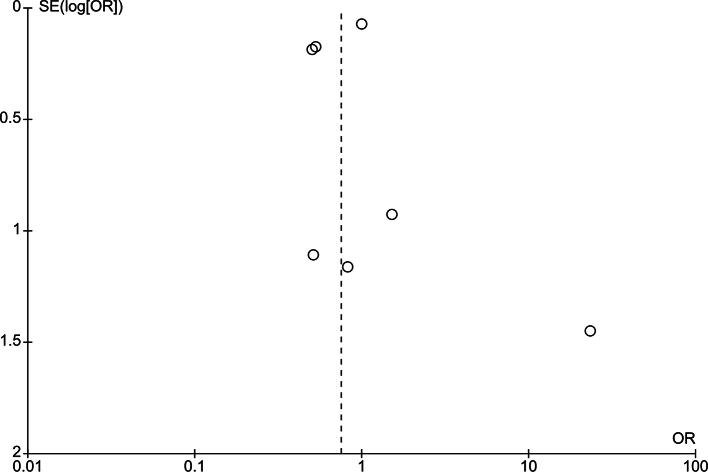
Forest plot – minor bleeding, follow-up.

**Supplementary Fig. S17:**
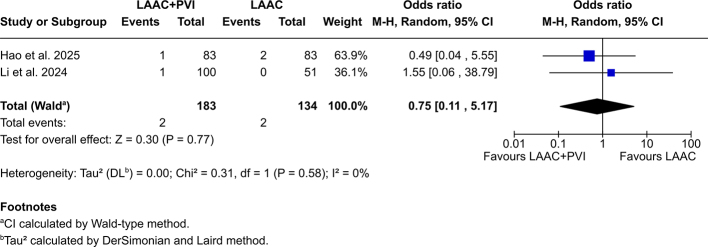
Funnel plot – minor bleeding, follow-up.

**Supplementary Fig. S18:**
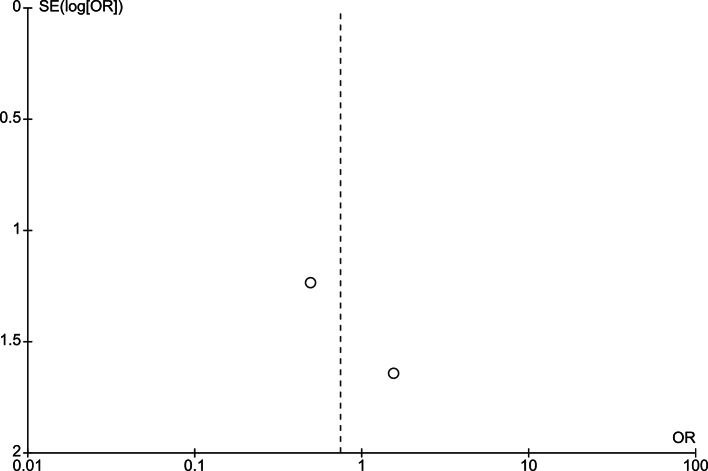
Forest plot – major bleeding, follow-up.

**Supplementary Fig. S19:**
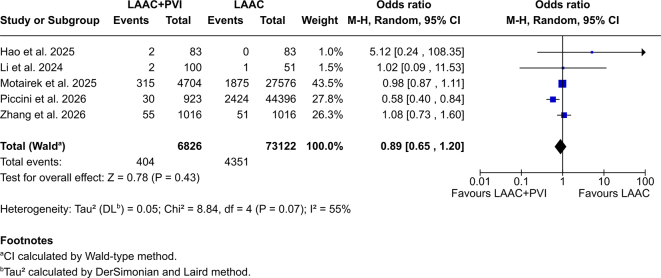
Funnel plot – major bleeding, follow-up.

Pericardial effusion requiring drainage was assessed as the primary safety endpoint and reported in ten studies. We found that patients undergoing concomitant LAAC+PVI were more likely to experience pericardial effusion with a need for drainage as compared to patients who underwent LAAC-only (OR = 1.72, 95% CI = 1.26–2.34, I^2^ = 0%; Fig. 5, Supplementary Fig. 6). For pericardial effusion without the need for drainage, which could be extracted from nine studies, there was no significant difference (OR = 1.62, 95% CI = 0.99–2.63, I^2^ = 0%; Fig. 6, Supplementary Fig. 7). Periprocedural systemic thromboembolisms were reported in eight studies, with no significant difference between groups (OR = 0.61, 95% CI = 0.23–1.63, I^2^ = 66%; Supplementary Figs. 8 and 9). Moreover, there were also no significant differences for periprocedural groin complications (OR = 1.26, 95% CI = 0.70–2.29, I^2^ = 0%; Supplementary Figs. 10 and 11), periprocedural death (OR = 1.03, 95% CI = 0.24–4.33, I^2^ = 61%; Supplementary Figs. 12 and 13), all-cause death during follow-up (OR = 0.76, 95% CI = 0.47–1.22, I^2^ = 77%; Supplementary Figs. 14 and 15), as well as minor (OR = 0.75, 95% CI = 0.11–5.17, I^2^ = 0%; Supplementary Figs. 16 and 17) and major bleeding (OR = 0.89, 95% CI = 0.65–1.20, I^2^ = 55%; Supplementary Figs. 18 and 19) at the end of follow-up.

### 3.4 Sensitivity analysis for study quality

**Supplementary Fig. S20:**
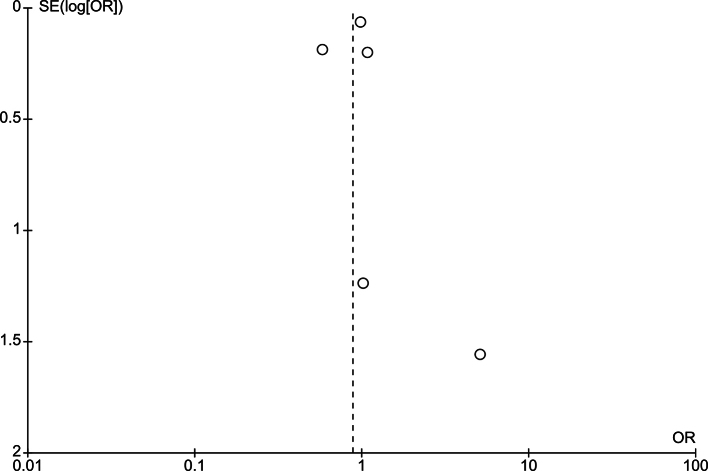
Forest plot – systemic thromboembolism, follow-up; sensitivity analysis.

**Supplementary Fig. S21:**
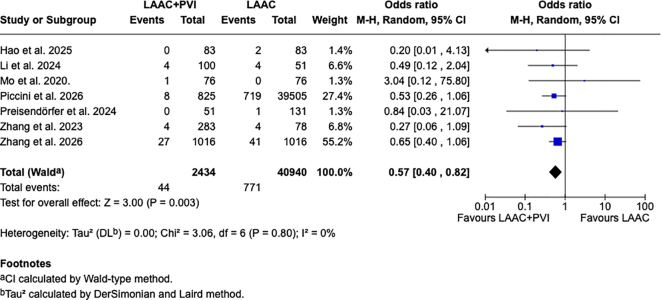
Funnel plot – systemic thromboembolism, follow-up; sensitivity analysis.

**Supplementary Fig. S22:**
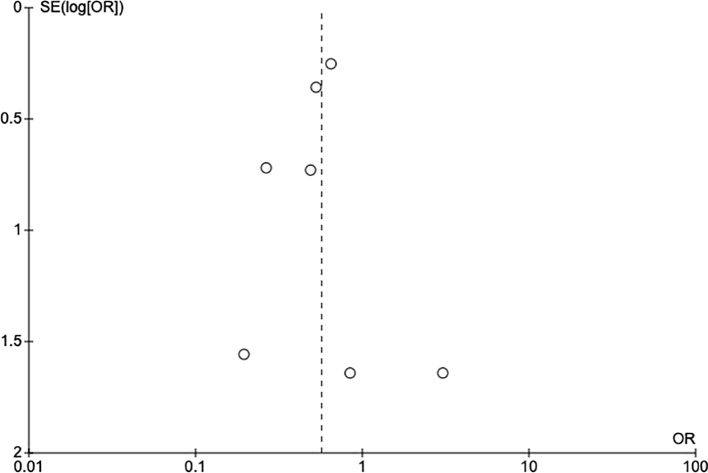
Forest plot – pericardial effusion requiring drainage; sensitivity analysis.

**Supplementary Fig. S23:**
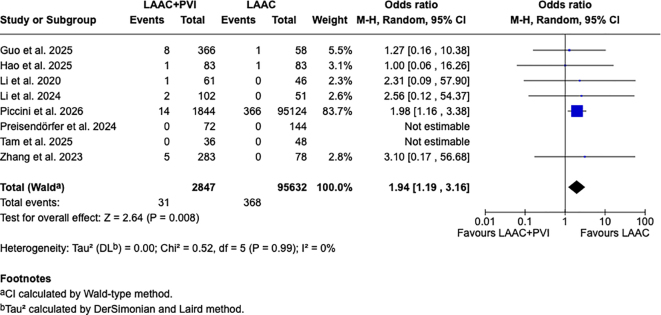
Funnel plot – pericardial effusion requiring drainage; sensitivity analysis.

Additionally, to test for result robustness, a sensitivity analysis restricting analyses only to studies rated “good” by the NIH quality tool was conducted for the four main outcomes. While no “fair”-quality studies were included in the analyses for periprocedural PDLs and PDLs at follow-up to begin with, the effect regarding systemic thromboembolism (OR = 0.57, 95% CI = 0.39–0.82, I^2^ = 0%; Supplementary Figs. 20 and 21) and pericardial effusion with a need for drainage (OR = 1.94, 95% CI = 1.19–3.16, I^2^ = 0%; Supplementary Figs. 22 and 23) even strengthened following their exclusion.

## 4 Discussion

In this comprehensive meta-analysis encompassing 15 trials and involving a total of 335,013 patients, we evaluated the safety and efficacy of combined LAAC+PVI compared with isolated LAAC in patients with atrial fibrillation. The key findings of this study are as follows: (1) Combined LAAC and PVI was associated with a significantly lower rate of systemic thromboembolism than LAAC alone. (2) At the same time, the combined approach carried a higher periprocedural risk of hemodynamically relevant pericardial effusion. (3) Peri-device leaks were less frequent at the time of implantation but (4) more frequent on follow-up transoesophageal echocardiography in the combined cohort.

The reduction in systemic thromboembolic events observed under the combined strategy is mechanistically plausible. LAAC addresses the dominant source of cardiogenic thrombi in non-valvular AF. [1], [2] In addition, restoration of sinus rhythm by PVI is thought to mitigate the contribution of atrial cardiomyopathy and recurrent arrhythmias to thromboembolic risk, and to reduce thrombus formation. [2], [13], [21], [24] Several recent large observational studies and registries have reported numerically lower rates of stroke, systemic embolism, or composite ischemic events in patients undergoing the combined procedure. These studies were not specifically powered for this endpoint, but hazard ratios were consistently below unity. [3], [15], [25] Remarkably, our findings diverge from those of a recent meta-analysis, which found no significant differences between combined and isolated LAAO across all assessed endpoints. This discrepancy is most plausibly explained by two factors. First, our search extended through May 2026 and captured three large registry-based studies published after Clemente's search cut-off, which together contributed the majority of patients in our thromboembolism and pericardial effusion with drainage analyses. Second, in contrast to our approach, the methods reported by Clemente et al. list peri-device leak only as a general outcome of interest, without specifying the timing of its assessment (periprocedural vs. follow-up). A pooling of peri-device leak data across different assessment time points must therefore be assumed, which, given the divergent, time-dependent pattern we observed, may have obscured a true effect. [6]

Combined LAAC and PVI was, however, associated with a higher periprocedural rate of cardiac tamponade. This finding is biologically plausible. The combined procedure entails longer left atrial dwell time and additional energy delivery. Depending on the chosen workflow, it may also require a second transseptal puncture. Each of these factors may contribute to mechanical or thermal injury of atrial structures. [3] The absolute event rates remained low. Nevertheless, the relative increase warrants careful patient selection, performance in experienced centres with established workflows for the combined procedure, and adherence to contemporary imaging and pericardial monitoring standards.

A particularly distinctive observation of the present analysis concerns the temporal behaviour of peri-device leaks. Periprocedural cumulative rates of PDL were significantly lower in the combined cohort. On follow-up imaging, however, the pattern was reversed. Significantly more leaks were observed in patients undergoing combined LAAC and PVI than in those undergoing isolated LAAC. This divergent dynamic is consistent with a previously described mechanism. Catheter ablation induces marked oedema of the left atrial ridge, which constrains the appendage ostium at the time of implantation. [26] The oedema then resolves during follow-up, thereby unmasking residual leak channels and reducing effective device compression over time. [5], [16], [23] This ablation-energy-dependent pattern was directly demonstrated in a recent single-centre cohort, in which peri-device leak increased markedly from 4.9% intraprocedurally to 23.3% at 45-day follow-up after radiofrequency ablation, whereas rates remained stable (5.1% to 5.1%) following pulsed-field ablation, consistent with reduced acute tissue oedema associated with PFA. [22] Whether pulsed-field ablation therefore confers an advantage over radiofrequency energy specifically in the context of concomitant LAAC and PVI, remains to be clarified in larger, multicentre cohorts. However, these observations have direct clinical implications. Assessment of seal at the time of implantation may overestimate the long-term sealing performance of LAAC devices in patients undergoing concomitant PVI. Structured post-procedural transoesophageal echocardiography at mid-term follow-up should therefore be regarded as an integral component of the combined procedure.

Taken together, these findings suggest that combined LAAC and PVI offers a meaningful thromboembolic benefit, at the cost of a distinct periprocedural risk profile that appears to differ from isolated LAAC. Given this trade-off, it may be reasonable to preferentially consider the one-stop approach in patients with a high thromboembolic risk relative to their individual bleeding and pericardial complication risk, while a staged approach may be preferable in patients with an elevated baseline risk of pericardial effusion or otherwise unfavourable anatomical or clinical characteristics. However, this consideration is hypothesis-generating and cannot be derived directly from the aggregate-level data underlying this meta-analysis and would require dedicated risk-stratified studies for validation. Careful patient selection, performance in dedicated centres, and structured post-procedural imaging follow-up are essential components of the combined procedure. Based on the divergent temporal pattern of peri-device leak observed in this analysis, post-procedural TEE surveillance extending beyond the immediate periprocedural period is warranted, although the included studies did not allow us to define an optimal or standardized surveillance interval. Moreover, future studies should address whether the increased periprocedural risk of pericardial effusion requiring drainage as well as the higher rate of peri-device leaks at follow-up associated with the concomitant procedure can be mitigated by a sequential approach, in which shorter individual procedure duration, reduced cumulative atrial manipulation, and resolution of post-ablation atrial oedema prior to device implantation may improve both periprocedural safety and long-term sealing performance.

### 4.1 Limitations

This meta-analysis is based predominantly on observational cohort studies and registries. These designs carry an inherent risk of confounding, despite the use of propensity score matching in several of the included studies. Notably, patients undergoing combined LAAC and PVI were significantly younger than those undergoing isolated LAAC (MD = −2.34, 95% CI −3.94 to −0.75), an imbalance driven predominantly by two studies.^17,23^ Whether this reflects a broader pattern of patient selection could not be fully resolved at the aggregate-data level. Similarly, because individual patient data were unavailable and procedural rhythm outcomes such as AT/AF recurrence were inconsistently reported, the extent to which the observed reduction in thromboembolic events reflects successful rhythm control by PVI, rather than an additive effect of LAAC itself, cannot be determined from the present data. In addition, the included studies used a heterogeneous mix of LAAC devices, ablation energy sources, procedural order, and follow-up protocols, which were frequently not reported, or not reported uniformly, precluding subgroup analyses stratified by ablation modality, procedural order, or study design. The timing of post-procedural TEE likewise varied considerably across studies, which may explain the substantial heterogeneity observed for peri-device leak at follow-up. A sensitivity analysis excluding studies of “fair” methodological quality did not attenuate the principal findings. Finally, our search excluded conference abstracts and grey literature, which may have introduced publication bias by underrepresenting smaller or negative studies. Given these limitations inherent to an aggregate-level meta-analysis of observational data, our findings should be regarded as hypothesis-generating, and randomized controlled trials are warranted to confirm them.

## 5 Conclusion

In this meta-analysis, combined LAAC and PVI was associated with a significantly lower rate of systemic thromboembolism compared with LAAC-only. This benefit came at the cost of a higher periprocedural rate of pericardial effusion requiring drainage. Furthermore, peri-device leaks showed a divergent temporal behaviour, being less frequent immediately post-procedure but more frequent on follow-up imaging in the combined cohort. Taken together, these findings refine the current understanding of the safety-efficacy balance of one-stop LAAC and PVI procedures and underscore the need for structured post-procedural imaging. Randomized controlled trials are warranted to confirm these findings.

The following are the supplementary data related to this article.

Captions Supplementary Figures.

Search strategy.

## CRediT authorship contribution statement

**Isabel Rattka:** Writing – review & editing, Writing – original draft, Investigation, Formal analysis, Data curation, Conceptualization. **Christine Poch:** Writing – review & editing, Writing – original draft, Visualization. **Tanja Mews:** Writing – review & editing, Data curation. **Constantin Friesacher:** Writing – review & editing, Software, Conceptualization. **Niklas Jurik:** Visualization, Validation, Methodology. **Eimo Martens:** Writing – review & editing, Resources, Formal analysis. **Karl-Ludwig Laugwitz:** Writing – review & editing, Validation, Supervision, Resources. **Alexander Steger:** Writing – review & editing, Writing – original draft, Conceptualization. **Manuel Rattka:** Writing – review & editing, Writing – original draft, Visualization, Validation, Software, Resources, Methodology, Investigation, Data curation, Conceptualization.

## Declaration of competing interest

Isabel Rattka received an educational grant from Boston Scientific. Manuel Rattka received honoraria for lectures and advisory board activities from Boston Scientific. Otherwise, there are no conflicts of interest.

## Data Availability

All relevant data are within the manuscript and supporting information files.
