# Supplementary material for: Efficacy and safety of concomitant left atrial appendage closure and pulmonary vein isolation compared to left atrial appendage closure only – A meta-analysis
Source: Int J Cardiol Heart Vasc. 2026 Aug 11;66:101988. doi: 10.1016/j.ijcha.2026.101988 (PMC13486899; doi:10.1016/j.ijcha.2026.101988)
Supplement: Supplementary file 1 [file mmc24.docx]

Supplementary Figure 1

Funnel plot – systemic thromboembolism, follow-up

Supplementary Figure 2

Forest plot – device related thrombus, follow-up

Supplementary Figure 3

Funnel plot – device related thrombus, follow-up

Supplementary Figure 4

Funnel plot – peri-device leaks, follow-up

Supplementary Figure 5

Funnel plot – peri-device leaks, periprocedural

Supplementary Figure 6

Funnel plot – pericardial effusion requiring drainage

Supplementary Figure 7

Funnel plot – pericardial effusion without drainage

Supplementary Figure 8

Forest plot – systemic thromboembolism, periprocedural

Supplementary Figure 9

Funnel plot – systemic thromboembolism, periprocedural

Supplementary Figure 10

Forest plot – groin complications, periprocedural

Supplementary Figure 11

Funnel plot – groin complications, periprocedural

Supplementary Figure 12

Forest plot – death, periprocedural

Supplementary Figure 13

Funnel plot – death, periprocedural

Supplementary Figure 14

Forest plot – death, follow-up

Supplementary Figure 15

Funnel plot – death, follow-up

Supplementary Figure 16

Forest plot – minor bleeding, follow-up

Supplementary Figure 17

Funnel plot – minor bleeding, follow-up

Supplementary Figure 18

Forest plot – major bleeding, follow-up

Supplementary Figure 19

Funnel plot – major bleeding, follow-up

Supplementary Figure 20

Forest plot – systemic thromboembolism, follow-up; sensitivity analysis

Supplementary Figure 21

Funnel plot – systemic thromboembolism, follow-up; sensitivity analysis

Supplementary Figure 22

Forest plot – pericardial effusion requiring drainage; sensitivity analysis

Supplementary Figure 23

Funnel plot – pericardial effusion requiring drainage; sensitivity analysis
