# Supplementary material for: Efficacy and safety of concomitant left atrial appendage closure and pulmonary vein isolation compared to left atrial appendage closure only – A meta-analysis
Source: Int J Cardiol Heart Vasc. 2026 Aug 11;66:101988. doi: 10.1016/j.ijcha.2026.101988 (PMC13486899; doi:10.1016/j.ijcha.2026.101988)
Supplement: Supplementary file 2 [file mmc25.docx]

**Supplementary Material 1 – Search Strategy**

An identical search string, based exclusively on free-text terms, was applied to all four databases (PubMed, MEDLINE via PubMed, Web of Science, and Scopus) through May 23rd, 2026.

**PubMed**

Search terms (free text):
("left atrial appendage closure" OR "left atrial appendage occlusion" OR "LAA closure" OR "LAA occlusion" OR "LAAC" OR "LAAO" OR "Watchman" OR "Amulet" OR "transcatheter left atrial appendage" OR "percutaneous left atrial appendage")
AND
("pulmonary vein isolation" OR "pulmonary vein ablation" OR "catheter ablation" OR "cryoablation" OR "cryoballoon" OR "pulsed field ablation" OR "radiofrequency ablation" OR "PVI" OR "PFA" OR "atrial fibrillation ablation")
AND
("atrial fibrillation" OR "AF" OR "AFib")
NOT
("surgical" OR "thoracoscopic" OR "epicardial" OR "ligation" OR "stapler" OR "AtriClip" OR "LARIAT" OR "thoracotomy" OR "mini-thoracotomy")

No MeSH terms were applied.

**MEDLINE (via PubMed)**

Search terms (free text): identical to the PubMed search string above.
No subject headings were applied.

**Web of Science**

Search terms (free text): identical to the PubMed search string above.
No subject headings were applied.

**Scopus**

Search terms (free text): identical to the PubMed search string above.
No subject headings were applied.
